# Blood glucose control and metabolic dysfunction-associated steatotic liver disease in people with type 1 diabetes

**DOI:** 10.1007/s40618-024-02333-2

**Published:** 2024-03-18

**Authors:** G. Della Pepa, R. Lupoli, M. Masulli, R. Boccia, R. De Angelis, S. Gianfrancesco, R. Piccolo, C. Rainone, A. A. Rivellese, G. Annuzzi, L. Bozzetto

**Affiliations:** 1 Department of Clinical Medicine and Surgery, Federico II University, Via Sergio Pansini 5, 80131 Naples, Italy; 2 Cardiometabolic Risk Unit, Institute of Clinical Physiology, National Research Council-CNR, Via Giuseppe Moruzzi 1, 56100 Pisa, Italy; 3 Department of Molecular Medicine and Medical Biotechnology, Federico II University, Via Sergio Pansini 5, 80131 Naples, Italy

**Keywords:** Metabolic dysfunction-associated steatotic liver disease, Glucose control, Type 1 diabetes, Fatty liver index, Hepatic steatosis index

## Abstract

**Purpose:**

Metabolic dysfunction-associated steatotic liver disease (MASLD) may have distinctive pathophysiological features in type 1 diabetes (T1D). We evaluated the independent role of blood glucose control on MASLD in T1D.

**Methods:**

In a cross-sectional study on 659 T1D adult patients, MASLD was assessed by the Fatty Liver Index (FLI) and the Hepatic Steatosis Index (HSI). Anthropometric, biochemical, and clinical parameters were retrieved from electronic records. Blood glucose control status was evaluated by dividing participants into subgroups according to the median value of HbA1c [7.6% (60 mmol/mol)], and this analysis was repeated excluding overweight/obese patients.

**Results:**

Patients with HbA1c above 7.6% (60 mmol/mol) showed significantly higher MASLD indices (HSI 38 ± 6 vs. 36 ± 5, p < 0.001; FLI 26 ± 26 vs.19 ± 19, p < 0.001), and higher proportions of MASLD identified by HSI (57 vs. 44%, p < 0.001) and FLI (14 vs. 7%, p < 0.001) than patients with HbA1c below 7.6% (60 mmol/mol). Similar results were obtained for HSI after the exclusion of overweight/obese patients. Stepwise linear regression analysis confirmed that HbA1c was independently associated with HSI (r = 0.496, p = 0.009) and FLI (r = 0.722, p = 0.007); waist circumference with HSI (r = 0.492, p < 0.001); and waist circumference (r = 0.700, p < 0.001), HDL cholesterol (r = 0.719, p < 0.001), and LDL cholesterol (r = 0.712, p < 0.001) with FLI.

**Conclusions:**

Blood glucose control is a main factor associated with MASLD in adults with T1D, also independently of overweight and obesity. Appropriate therapeutic strategies focused on tight blood glucose control may also be needed for the prevention and treatment of MASLD in T1D.

## Introduction

Metabolic dysfunction-associated steatotic liver disease (MASLD) formerly named non-alcoholic fatty liver disease or NAFLD [1] is the most common liver disease worldwide, affecting 20–30% of the general population [2]. It includes different histopathological abnormalities ranging from triglyceride accumulation in the hepatocytes (liver steatosis) to metabolic dysfunction-associated steatohepatitis (MASH), liver fibrosis, and advanced cirrhosis [3].

MASLD shares with type 2 diabetes (T2D) a strict association with insulin-resistance and all components of the metabolic syndrome, such as overweight/obesity, dyslipidaemia and hypertension [4, 5]. Consistent with this common pathophysiological milieu, almost the totality of people with T2D have MASLD [6, 7]. However, it appears to exist a bidirectional relationship between MASLD and T2D. Indeed, MASLD may act as a trigger for glucose metabolism disruption, and hyperglycaemia may in turn sustain ectopic fat accumulation [8].

Obesity and related metabolic alterations are a growing concern also in people with type 1 diabetes (T1D) [9], being a risk factor for MASLD also in this population [10]. However, MASLD in T1D seems to have pathophysiological peculiarities whose understanding could help in disentangling the complex relationship between diabetes and MASLD. One of these factors is hyperglycaemia not associated with endogenous hyperinsulinemia and /or insulin resistance. The hyperglycaemic status may play a relevant role by acting on the onset and progression of MASLD through different mechanisms [11, 12]. Few studies have addressed this specific aspect suggesting that blood glucose control may play a role [13–16], but it has not been investigated whether this role is independent of other confounding factors, in particular overweight/obesity that are increasing in adult people with T1D.

From a clinical point of view, the identification of the main factors associated with MASLD in T1D, particularly the early forms that are potentially reversible (i.e. liver steatosis), would be of great interest and could aid patients to benefit from therapeutic intervention such as lifestyle modification or tight glucometabolic control [17], considering also that the presence of MASLD in T1D is associated with a poorer metabolic profile and a higher prevalence of microvascular and macrovascular complications [10, 18, 19].

Therefore, the aim of our study was to assess the possible association of glucose control independently of other confounding factors with MASLD, evaluated by indirect indices, in a large population of adult patients with T1D.

## Materials and methods

### Study design and population

We performed a cross-sectional, single-center study on patients with T1D who carried out the yearly diabetes complications’ assessment at the Diabetes Unit of Federico II University of Naples from 2010 to 2021. The medical records of each patient’s most recent visit were reviewed to collect clinical and biochemical variables. Adult patients (18–80 years old) with T1D of both genders and a diabetes duration of at least 1 year were included in the present analysis. We have excluded patients with any acute or chronic hepatic disease, and a history of alcohol intake exceeding 30 g/day in men and 20 g/day in women. T1D was defined by the use of insulin in combination with either the presence of anti-GAD or anti-islet cell auto-antibodies, and/or a clearly documented diagnosis of T1D [17].

The study protocol—performed in accordance with the Declaration of Helsinki—was approved by the Ethics Committee of Federico II University. All participants provided written informed consent to using their clinical and laboratory data and being included in the study.

### Measurements

Body weight, height, and waist circumference were measured by standard procedures, and body mass index (BMI) was calculated as weight (kg) / height (m^2^). All participants underwent a complete screening of chronic complications according to a standardized protocol including clinical examination and dilated eye exam for diabetic retinopathy screening. Nephropathy was assessed by urinary albumin excretion rate, serum creatinine, and estimated glomerular filtration rate (eGFR). Autonomic nerve function was assessed by cardiovascular reflex tests: parasympathetic function by heart rate variability through a deep breathing test (beat-to-beat variation), and sympathetic function by blood pressure response to standing. Peripheral neuropathy was assessed by bilateral vibration perception, tactile perception (Semmes–Weinstein monofilament), and ankle reflex.

Blood samples were obtained in the morning after an overnight fast. All biochemical analyses were performed at the outpatient laboratory of the Federico II University of Naples, using standard procedures. Total and HDL-cholesterol were measured by standard methods. LDL-cholesterol was calculated according to the Friedewald equation only for triglyceride values < 400 mg/dl. Glycated hemoglobin (HbA1c) was measured by high liquid performance chromatography standardized according to IFCC. Liver enzymes—*i.e.*, aspartate aminotransferase (AST), alanine aminotransferase (ALT), and gamma-glutamyl-transpeptidase (GGT)—were measured by colorimetric methods. Daily insulin dose was calculated as the sum of all insulin doses injected per day divided by body weight.

Indirect indices of MASLD were calculated according to the following formulas:

- Hepatic Steatosis Index (HSI): 8 × ALT/AST ratio + BMI (+ 2, if diabetes mellitus; + 2, if female), with values < 30 ruling out and values > 36 ruling in steatosis [20].
- Fatty Liver Index (FLI): (e ^0.953 × log (triglycerides) + 0.139 × BMI + 0.718 × log (GGT) + 0.053 × waist circumference − 15.745^) / (1 + e ^0.953 × log (triglycerides) + 0.139 × BMI + 0.718 × log (GGT) + 0.053 × waist circumference − 15.745)^ × 100, with values < 30 ruling out and values ≥ 60 ruling in steatosis [21].

Insulin sensitivity was evaluated as the estimated glucose disposal rate (eGDR):

- eGDR = 21.158 − (0.09 × waist circumference) − (3.407 × hypertension (yes = 1/no = 0) − (0.551 × HbA1c) [22], where hypertension is 1 if blood pressure ≥ 140/90 mm Hg and/or patient takes antihypertensive drugs. Lower eGDR values correspond to higher insulin resistance, with a cut off value < 9.65 considered suggestive of insulin resistance syndrome.

### Statistical analysis

Data are presented as mean ± standard deviation for continuous variables or frequencies and percentages for categorical variables. Continuous variables were compared between groups using the t-test for normally distributed variables, Mann–Whitney-*U* test for skewed variables, and χ^2^ test or Fisher’s exact test for categorical variables. Due to baseline differences in gender distribution between groups, the characteristics of the participants were compared using ANCOVA general linear model taking the variable of interest (i.e., age, BMI, waist circumference, etc.) as dependent variable, MASLD-status as fixed factor, and gender as covariate. To explore the possible impact of blood glucose control status, participants were divided into subgroups according to the median value of HbA1c and this analysis was repeated excluding overweight/obese patients. A stepwise linear regression analysis was performed to assess the association between variables of interest and HSI and FLI. A *p* value < 0.05 was considered statistically significant. Statistical analysis was performed using SPSS software 26.0 (SPSS/PC; IBM, Armonk, NY, USA).

## Results

### *Characteristics of the participants according to* MASLD *status*

Anthropometric, biochemical, and clinical parameters of the whole population (n = 659 patients) are summarized in Table 1. On average, age was 37 years, BMI 25.4 kg/m^2^, HbA1c 7.8% (62 mmol/mol), and duration of diabetes 20 years. In Table 2**,** data are reported according to MASLD status. As for HSI, 51% of the participants were above the cut off value of 36. These patients were older, more likely to be male, overweight/obese and with a higher waist circumference, and had a longer duration of diabetes than participants with HSI < 30. They also had higher plasma LDL-cholesterol, lower insulin sensitivity, a moderately lower eGFR, and more use of lipid lowering drugs (23 vs. 0.04%) and retinopathy (26 vs. 7%). An FLI above the cut off value of 60 was present in 10% of the participants. These patients were older, more likely to be male, overweight/obese and with a higher waist circumference, and had a longer duration of diabetes than participants with FLI < 30. They also had a worse glucose control and plasma lipid profile, lower insulin sensitivity, lower eGFR, and more use of lipid lowering drugs (37 vs. 12%) and microvascular complications.

**Table 1:** Anthropometric, clinical, and biochemical data of the T1D study participants

	n = 659
Women/men *n* (%)	319/340 (48/52)
Age (years)	37 ± 13
BMI (kg/m^2^)	25.4 ± 4
Participants with BMI ≥ 30* n* (%)	68 (10)
Waist circumference (cm)	86 ± 13
Duration of diabetes (years)	20 ± 12
HbA_1c_ (%)	7.8 ± 1.2
HbA_1c_ (mmol/mol)	62 ± 13
Plasma triglycerides (mg/dl)	81 ± 54
HDL-cholesterol (mg/dl)	61 ± 16
LDL-cholesterol (mg/dl)	103 ± 30
Uric acid (mg/dl)	4.1 ± 3.9
Gamma glutamyl transpeptidase (U/l)	19 ± 19
C-reactive protein (mg/dl)	0.4 ± 0.5
Estimated Glomerular Filtration Rate (ml/min/1.73 m^2^)	96 ± 19
Daily insulin dose (IU/body weight)	0.60 ± 0.25
Estimated glucose disposal rate (mg/kg/min)	8.3 ± 2.3
Lipid lowering drugs users *n* (%)	118 (18)
Nephropathy* n* (%)	79 (12)
Retinopathy* n* (%)	138 (21)
Neuropathy *n* (%)	84 (13)

Data are means (SD) or frequency (percentage)

*BMI* body mass index; *HbA1c* glycated hemoglobin; *HDL* high density lipoproteins; *LDL* low density lipoproteins

**Table 2:** Anthropometric, clinical, and biochemical data of the T1D study population according to MASLD status identified by HSI and FLI cut-offs

	HSI < 30 (n = 28; 4%)	HSI > 36 (n = 337; 51%)	*p*^†^	FLI < 30 (n = 474; 72%)	FLI ≥ 60 (n = 68; 10%)	*p*^†^
Women/men *n* (%)	17/11 (61/39)	139/198 (41/59)	**0.049**	264/210 (56/34)	22/46 (32/68)	** < 0.001**
Age (years)	31 ± 11	39 ± 13	** < 0.001**	35 ± 12	44 ± 13	** < 0.001**
BMI (kg/m^2^)	19.8 ± 1.4	27.9 ± 3.5	** < 0.001**	24 ± 3	32 ± 4	** < 0.001**
BMI ≥ 30* n* (%)	0 (0)	68 (20)	**0.004**	5 (1)	43 (63)	** < 0.001**
Waist circumference (cm)	69 ± 13	93 ± 12	** < 0.001**	80 ± 9	107 ± 11	** < 0.001**
Duration of diabetes (years)	15 ± 10	22 ± 12	**0.006**	19 ± 11	23 ± 11	**0.001**
HbA_1c_ (%)	7.5 ± 1.6	7.9 ± 1.2	0.084	7.6 ± 1.2	8.3 ± 1.5	** < 0.001**
HbA_1c_ (mmol/mol)	58 ± 18	63 ± 13	0.084	60 ± 13	67 ± 17	** < 0.001**
Plasma triglycerides (mg/dl)	72 ± 30	90 ± 58	0.194	67 ± 27	158 ± 98	** < 0.001**
HDL-cholesterol (mg/dl)	63 ± 15	58 ± 16	0.296	63 ± 16	52 ± 13	** < 0.001**
LDL-cholesterol (mg/dl)	92 ± 26	106 ± 30	**0.022**	100 ± 29	116 ± 30	** < 0.001**
Uric acid (mg/dl)	3.7 ± 1.2	4.2 ± 2.1	0.486	3.9 ± 4.3	4.6 ± 1.4	0.387
Gamma glutamyl transpeptidase (U/l)	15 ± 10	21 ± 19	0.196	15 ± 14	36 ± 34	** < 0.001**
C-reactive protein (mg/dl)	0.3 ± 0.1	0.4 ± 0.5	0.388	0.4 ± 0.4	0.5 ± 0.4	0.112
Estimated glomerular filtration rate (ml/min/1.73 m^2^)	102 ± 19	94 ± 19	**0.020**	97 ± 18	89 ± 23	** < 0.001**
Daily insulin dose (IU/kg body weight)	0 .63 ± 0.29	0.59 ± 0.23	0.383	0.6 ± 0.2	0.6 ± 0.3	0.278
Estimated glucose disposal rate (mg/kg/min)	10.6 ± 1.5	7.4 ± 2.3	** < 0.001**	9.2 ± 1.7	5.1 ± 2.3	** < 0.001**
Lipid lowering drugs users *n* (%)	1 (0.04)	78 (23)	**0.017**	55 (12)	25 (37)	** < 0.001**
Nephropathy* n* (%)	2 (7)	46 (14)	0.321	49 (10)	19 (28)	** < 0.001**
Retinopathy* n* (%)	2 (7)	89 (26)	**0.029**	81 (17)	33 (49)	** < 0.001**
Neuropathy *n* (%)	2 (7)	60 (18)	0.113	39 (8)	30(44)	** < 0.001**

Data are means (SD) or frequency (percentage)

Significant *p* values are reported in bold

*BMI* body mass index; *HbA1c* glycated hemoglobin; *HDL* high density lipoproteins; *LDL* low density lipoproteins

^†^Adjusted by gender

### Characteristics of the participants and MASLD status according to blood glucose control

Anthropometric, biochemical, clinical parameters, and MASLD status of the patients according to the median value of HbA1c [above or below 7.6% (60 mmol/mol)] are shown in Table 3.

**Table 3:** Anthropometric, clinical, and biochemical data, and MASLD status of the T1D study population stratified according to the median HbA1c

	HbA_1c_ < 7.6% (n = 304)	HbA_1c_ ≥ 7.6% (n = 350)	*p*^†^
Women/men *n* (%),	131/173 (43/57)	187/163 (53/47)	**0.010**
Age (years)	37 ± 13	37 ± 13	0.792
BMI (kg/m^2^)	25 ± 4	26 ± 4	**0.001**
BMI ≥ 30* n* (%)	19 (6)	48 (14)	**0.001**
Waist circumference (cm)	85 ± 12	87 ± 14	**0.003**
Duration of diabetes (years)	20 ± 12	20 ± 12	0.795
HbA_1c_ (%)	6.8 ± 0.5	8.5 ± 1.0	** < 0.001**
HbA_1c_ (mmol/mol)	51 ± 5.6	69 ± 11	** < 0.001**
Plasma triglycerides (mg/dl)	74 ± 41	88 ± 63	**0.001**
HDL-cholesterol (mg/dl)	61 ± 15	61 ± 17	0.285
LDL-cholesterol (mg/dl)	99 ± 28	107 ± 31	**0.001**
Uric acid (mg/dl)	4.1 ± 1.2	4.2 ± 5.3	0.667
HSI	36 ± 5	38 ± 6	** < 0.001**
< 30 *n* (%)	17 (6)	11 (3)	**0.032**
> 36 *n* (%)	136 (44)	200 (57)	** < 0.001**
FLI	19 ± 19	26 ± 26	** < 0.001**
< 30 *n* (%)	238 (78)	232 (66)	** < 0.001**
≥ 60 *n* (%)	20 (7)	48 (14)	** < 0.001**
Gamma glutamyl transpeptidase (U/l)	18 ± 21	19 ± 17	0.236
C-reactive protein (mg/dl)	0.3 ± 0.3	0.4 ± 0.6	0.155

Data are means (SD) or frequency (percentage)

Significant *p* values are reported in bold

*BMI* body mass index; *HbA1c* glycated hemoglobin; *HDL* high density lipoproteins; *LDL* low density lipoproteins; *HSI* hepatic steatosis index; *FLI* fatty liver index

^†^Adjusted by gender

Patients with HbA1c above 7.6% (60 mmol/mol) were more likely to be female, overweight/obese, and had a higher waist circumference, a higher values of plasma triglycerides and LDL-cholesterol. In these patients, HSI values and the proportion of patients with MASLD identified by HSI (57 vs. 44%) were significantly higher. Similarly, FLI values and the proportion of patients with MASLD identified by FLI (14 vs. 7%) were significantly higher in patients with HbA1c above 7.6% (60 mmol/mol).

### MASLD according to blood glucose control in the normal-weight participants

To evaluate the association of glucose control with MASLD independently of overweight/obesity, analyses were performed in the subgroup of patients with BMI < 25 kg/m^2^ divided according to the median value of HbA1c (Table 4). HSI levels and the proportion of patients with MASLD (22 vs. 13%) were significantly higher in the patients with HbA1c above 7.6% (60 mmol/mol) than those with HbA1c below 7.6% (60 mmol/mol), with no significant differences in the other anthropometric and metabolic parameters between the two groups. No significant differences in FLI were observed, likely due to its low levels in both groups (Table 4). Stepwise linear regression analysis confirmed that HbA1c was independently associated with MASLD values identified by HSI (r = 0.496; p = 0.009) and FLI (r = 0.722; p = 0.007). Moreover, HSI was associated with waist circumference (r = 0.492; p < 0.001) and FLI with waist circumference (r:0.700; p < 0.001), HDL-cholesterol (r = 0.719; p < 0.001), and LDL cholesterol (r = 0.712; p < 0.001).

**Table 4:** Anthropometric, clinical, and biochemical data, and MASLD status according to the median HbA_1c_ in the normal-weight subgroup of T1D participants

	HbA_1c_ < 7.6% (n = 160)	HbA_1c_ ≥ 7.6% (n = 157)	*p*^†^
Women/men *n* (%)	79/81 (49/51)	95/62 (61/39)	**0.030**
Age (years)	35 ± 13	35 ± 12	0.816
Body mass index (kg/m^2^)	22 ± 2	22 ± 2	0.565
Waist circumference (cm)	78 ± 8	77 ± 9	0.429
Duration of diabetes (years)	17 ± 11	17 ± 11	0.951
HbA_1c_ (%)	6.8 ± 0.5	8.5 ± 1.1	** < 0.001**
HbA_1c_ (mmol/mol)	51 ± 5.5	69 ± 12	** < 0.001**
Plasma triglycerides (mg/dl)	67 ± 35	70 ± 29	0.381
HDL-cholesterol (mg/dl)	63 ± 15	62 ± 18	0.836
LDL-cholesterol (mg/dl)	97 ± 28	102 ± 32	0.191
Uric acid (mg/dl)	3.8 ± 1.0	4.2 ± 7.0	0.509
HSI	33 ± 3	34 ± 3	**0.010**
< 30 *n* (%)	17 (12)	11 (6)	0.074
> 36 *n* (%)	21 (13)	34 (22)	**0.025**
FLI	8.7 ± 8.5	9.2 ± 9.0	0.272
< 30 *n* (%)	154 (96)	150 (96)	0.826
≥ 60 *n* (%)	1 (0.7)	0 (0)	0.330
Gamma glutamyl transpeptidase (U/l)	18 ± 22	18 ± 13	0.900
C-reactive protein (mg/dl)	0.3 ± 0.3	0.4 ± 0.4	0.788

Data are means (SD) or frequency (percentage)

Significant *p* values are reported in bold

*HbA1c* glycated hemoglobin; *HDL* high density lipoproteins; *LDL* low density lipoproteins; *HSI* hepatic steatosis index; *FLI* fatty liver index

^†^Adjusted by gender

## Discussion

The main novel finding of this large cross-sectional study is that, in patients with T1D, a better blood glucose control was associated with lower MASLD indices and a lower prevalence of MASLD. This association was independent of other anthropometric and metabolic determinants, was confirmed by both indices utilized (HSI and FLI), and was independent of obesity/overweight as it was also observed in the subgroup of participants with normal weight. Furthermore, the association was clinically relevant because the normal-weight patients with T1D with worse blood glucose control showed almost double the risk of MASLD compared to those with better glucose control.

From a clinical point of view, the identification of glucose control as an independent factor associated with liver fat accumulation, a potentially reversible manifestation of MASLD, could further motivate clinicians and patients to pursue a tighter glucose control, as both MASLD and scarce blood glucose control may independently lead to a higher prevalence of microvascular and macrovascular complications [10, 18, 19].

The determinants of MASLD in T1D might differ from those typical of obesity, metabolic syndrome, and T2D. Our results show that in adults with T1D a primary role in the onset and progression of MASLD might be blood glucose control. Literature data concerning the independent role of glucose control are few, and performed in populations with different anthropometric characteristics, age, and sample size.

In epidemiological studies, the presence of MASLD in adults with T1D was associated with poor blood glucose control together with other factors, such as age, duration of diabetes, modalities of subcutaneous insulin administration, and microvascular complications [10, 13, 18, 19, 23]. In children with T1D only glucose control significantly correlated with MASLD, and the improvement in glycated hemoglobin over 6 months promoted a reduction in liver fat in 60% of patients [16]. In young individuals with T1D, poor glucose control was the major risk factor for MASLD evaluated by ultrasonography [24]; while, in a similarly young cohort, the major determinants of MASLD, evaluated by FibroScan, were glycated haemoglobin, gender, BMI, and HDL-cholesterol [25].

Glucose control might impact MASLD by favouring the accumulation of triglycerides within the hepatocytes through the activation and upregulation by hyperglycaemia of key transcriptional factors involved in de novo lipogenesis, such as carbohydrate responsive element binding protein and sterol regulatory element binding protein-1c [11, 12]; furthermore, in animal models, hyperglycaemic conditions over-express glucose transporter 2 that may contribute to liver fat accumulation by an overflow of glucose in the hepatocyte [26].

Beyond the independent role of blood glucose control, in our study we confirmed the association between MASLD and different features of metabolic syndrome [4]. This relationship, likely driven by insulin resistance [8], is clinically relevant considering that in the last decade the prevalence of features of metabolic syndrome has also increased in T1D [27]. In line with this, in our population, patients with MASLD showed a higher prevalence of dyslipidemia or use of lipid lowering drugs [18, 19, 28], and also confirmed the association of MASLD with age, duration of diabetes, and microvascular complications [18, 19, 28].

While the prevalence of MASLD in T2D is well estimated, ranging from 55 to 70% [7], in T1D it widely ranges from 5 to 55% according to different diagnostic tools and population characteristics [29]. A comprehensive meta-analysis found a prevalence of 22% in adults with T1D [30]. In our cohort, MASLD prevalence was different according to using HSI or FLI. When detected by HSI, it was 51%, which is in line with epidemiological data coming from Italian cohorts of patients with T1D and MASLD detected by ultrasonography [28, 31]; it was, instead, 10%, when detected by FLI. This difference could reflect the presence of diabetes status as a component of the HSI algorithm, possibly overestimating the contribution of insulin resistance to MASLD. On the other hand, it should be considered that FLI may identify more severe degrees of fatty liver [32], and its lower sensitivity [21] might have led to an underestimation of the MASLD prevalence.

Our study had some limitations. First, the relationship between blood glucose control and MASLD in normal-weight patients, as well as the association with all the other factors, cannot be considered causal because of the cross-sectional study design. Therefore, the possible bidirectional relationship where hyperglycemia promotes liver fat accumulation and, conversely, MASLD contributes to worsening glycemic control should be considered [33]. Second, potential confounding factors, such as dietary habits and physical activity level, were not examined. Furthermore, although being a large sample size cohort, the study population was from a tertiary care center, which makes it difficult to rule out selection bias. Finally, MASLD was detected by indirect indices. In this regard, although liver biopsy represents the gold standard for the diagnosis of MASLD, it is not feasible in epidemiological studies. Among several indices, based on non-invasive measures and easily performed in clinical practice, proposed for the diagnosis of MASLD [34, 35], HSI and FLI were used in several epidemiological studies investigating the presence of MASLD in patients with T1D [32, 36–39]. With this regard, these indices were also validated against Magnetic Resonance Imaging in patients with T1D, showing a good sensitivity [31]. Of note, HSI has been used in a very recent study performed in a large Italian population of adult patients with T1D showing similar features to our population [39].

These limitations are compensated by several strengths: a large sample size, a well-defined population of patients with T1D routinely observed in clinical practice, the collection of clinical data according to standard methods, and the biochemical measurements performed in a centralized laboratory.

## Conclusions

In our study, we show that blood glucose control is a main factor associated with MASLD in adults with T1D, also independently of overweight and obesity. This finding strongly indicates that appropriate therapeutic strategies focused on tight blood glucose control are needed in T1D even for the prevention and treatment of the early stages of MASLD.

## Data Availability

The data associated with the study are available from the corresponding author on reasonable request.

## Abbreviations

AST: Aspartate aminotransferase
ALT: Alanine aminotransferase
BMI: Body mass index
eGDR: Estimated glucose disposal rate
GGT: Gamma glutamyl transpeptidase
HbA1c: Glycated hemoglobin
FLI: Fatty liver index
HSI: Hepatic steatosis index
MASH: Metabolic dysfunction-associated steatohepatitis
MASLD: Metabolic dysfunction-associated steatotic liver disease
T1D: Type 1 diabetes
